# Pulse-spray catheter-directed thrombolysis with monteplase for IVC filter-associated thrombosis after massive pulmonary embolism requiring VA-ECMO: a case report

**DOI:** 10.1093/ehjcr/ytag202

**Published:** 2026-03-13

**Authors:** Atsushi Sato, Naoya Niwa, Masafumi Tsurumi, Tomohiko Ono, Keisuke Matsumura

**Affiliations:** Department of Cardiology, NHO Saitama Hospital, 2-1 Suwa, Wako, Saitama 351-0102, Japan; Department of Cardiology, NHO Saitama Hospital, 2-1 Suwa, Wako, Saitama 351-0102, Japan; Department of Cardiology, NHO Saitama Hospital, 2-1 Suwa, Wako, Saitama 351-0102, Japan; Department of Cardiology, NHO Saitama Hospital, 2-1 Suwa, Wako, Saitama 351-0102, Japan; Department of Cardiology, NHO Saitama Hospital, 2-1 Suwa, Wako, Saitama 351-0102, Japan

**Keywords:** Case report, Pulmonary embolism, Deep vein thrombosis, Inferior vena cava filter, Catheter-directed thrombolysis, Pulse-spray method, Monteplase

## Abstract

**Background:**

Heparin-based anticoagulation is the first-line therapy for pulmonary embolism and deep vein thrombosis. However, when thrombus progresses despite adequate anticoagulation, placement of an inferior vena cava (IVC) filter may be considered. Thrombus extension following IVC filter placement may complicate retrieval and necessitate thrombolytic therapy, which carries a risk of bleeding. Here, we report a case in which catheter-directed thrombolysis (CDT) using monteplase was successfully performed for thrombus progression after IVC filter placement without any haemorrhagic complications.

**Case summary:**

A 33-year-old man presented with sudden chest pain and dyspnoea. He developed pulseless electrical activity (PEA) shortly after arrival and was resuscitated with veno-arterial extracorporeal membrane oxygenation (ECMO). Pulmonary arteriography and CT revealed bilateral pulmonary artery thrombi. Anticoagulation therapy was initiated, and ECMO was discontinued on Day 8 to stabilize his haemodynamics. However, pulmonary embolism recurred and deep vein thrombosis progressed despite adequate heparinization, necessitating placement of an IVC filter and ultimately CDT using monteplase via the pulse-spray method. The thrombus burden resolved without significant bleeding.

**Discussion:**

If thrombus progression is observed after IVC filter placement, pulse-spray CDT using monteplase may help prevent complications, such as difficulty in filter retrieval and development of post-thrombotic syndrome, without any bleeding, particularly when urokinase is unavailable.

Learning points

- In cases of pulmonary embolism, complete reperfusion should be achieved even with VA-ECMO support, and vigilance for recurrence is essential despite anticoagulation with heparin.
- Catheter-directed thrombolysis (CDT) enables targeted thrombus dissolution by delivering thrombolytic agents directly into the clot, minimizing systemic exposure and reducing bleeding risk.
- The pulse-spray technique may further enhance efficacy by promoting deeper intrathrombus drug penetration.

## Background

Massive pulmonary embolism (PE) with circulatory collapse carries a high mortality risk. Veno-arterial extracorporeal membrane oxygenation (VA-ECMO) can provide haemodynamic support during resuscitation. Anticoagulant therapy with heparin is the standard initial treatment for PE and deep vein thrombosis (DVT). In cases where the thrombus enlarges despite adequate heparinization, placement of an inferior vena cava (IVC) filter may be considered. However, retrieval of an IVC filter can become challenging if the thrombus continues to grow post-placement.^1^

Urokinase is the preferred systemic thrombolytic therapy for patients with PE and DVT but has been in short supply worldwide as a consequence of production difficulties that started during the COVID-19 pandemic. Owing to the ongoing unavailability of urokinase, we have been using monteplase, which is associated with an increased risk of bleeding.^2^ However, catheter-directed thrombolysis (CDT) allows for localized delivery of thrombolytic agents, offering the advantage of fewer adverse effects than would be expected via the systemic route.^3^

This report describes a case in which CDT administered by the pulse-spray method was effective in managing the bleeding risk in a patient with massive PE and DVT in whom an IVC filter had become filled with thrombus.

## Summary figure

**Figure:**
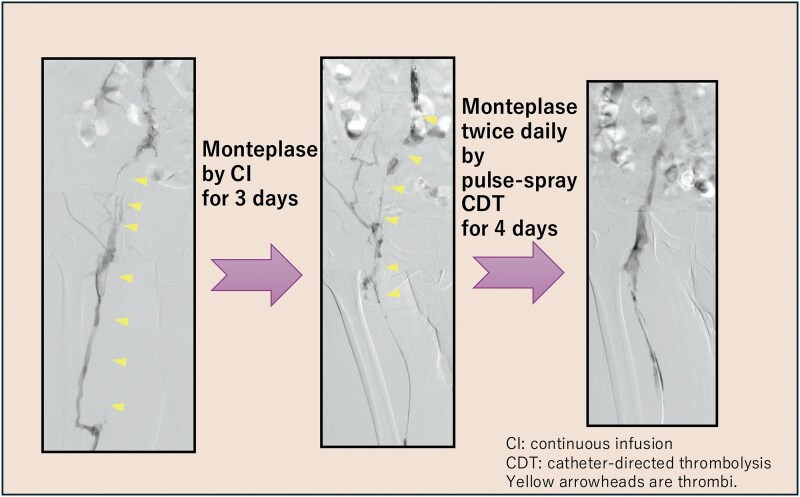


## Case summary

**Figure 1:**
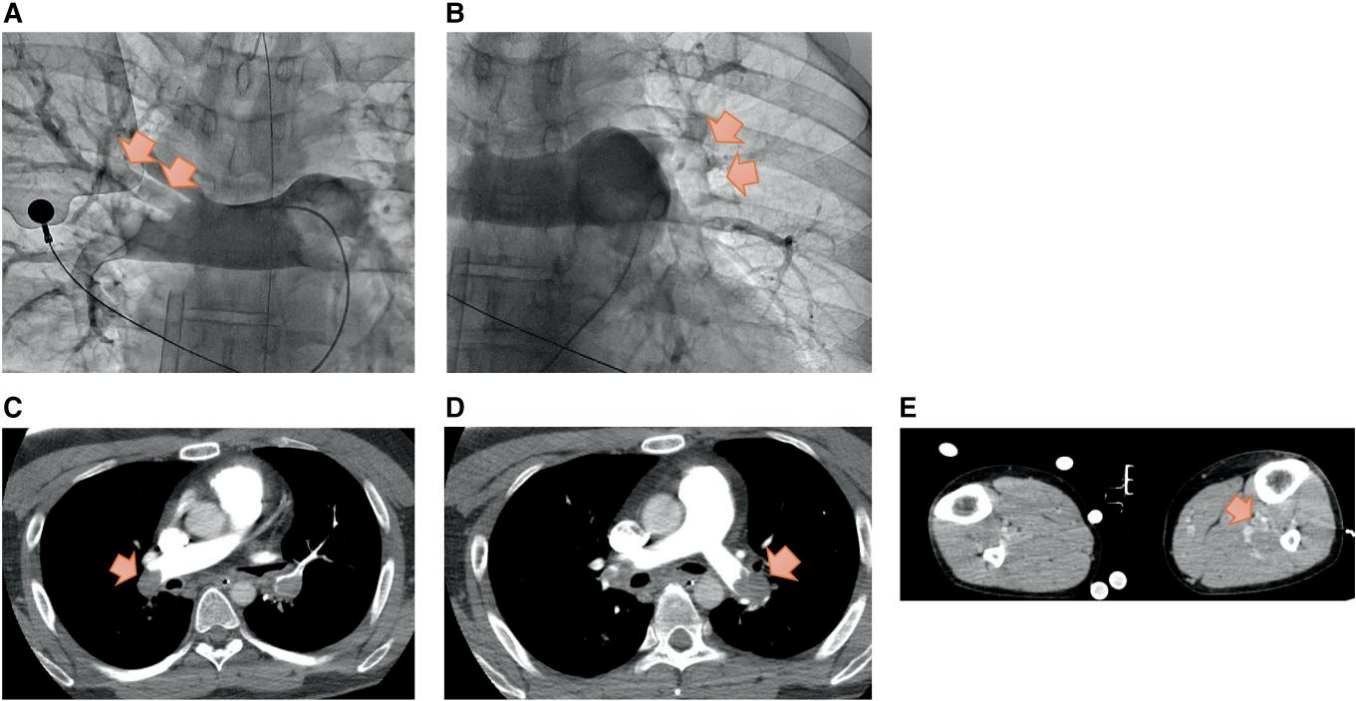
Imaging findings on admission. (*A*, *B*) Pulmonary angiography images and (*C–E*) computed tomography images showed thrombi in the proximal portions of both pulmonary arteries and in the posterior tibial and fibular veins (arrows).

A 33-year-old man presented via emergency medical services with complaints of chest pain and dyspnoea. The patient developed pulseless electrical activity (PEA) immediately after hospital arrival, before monitoring could be established, which prompted immediate initiation of cardiopulmonary resuscitation (CPR) and endotracheal intubation. Transthoracic echocardiography revealed right ventricular enlargement without pericardial effusion, raising suspicion for PE and prompting initiation of VA-ECMO. The PESI score was 173 and the sPESI score was 2, corresponding to class V and indicating a very high risk. VA-ECMO was initiated in the catheterization laboratory via percutaneous cannulation of the right femoral artery and vein under ultrasound guidance, after confirming by ultrasound that no thrombus was present in the femoral vein. Pulmonary angiography using DSA revealed thrombi in the main trunks of both pulmonary arteries, leading to a diagnosis of massive (collapse-type) PE. The mean pulmonary artery pressure was 63 mmHg. Although a catheter-based aspiration device for thrombus removal was considered potentially effective, its clinical use had not been approved in our hospital. Contrast-enhanced computed tomography (CT) revealed thrombi in the left posterior tibial and peroneal veins, confirming concomitant DVT (*Figure 1*). Laboratory investigations showed elevated D-dimer (118 µg/mL) and lactate (16 mmol/L). No apparent risk factors, predisposing conditions, or provoking triggers were identified. The patient had been previously healthy, with no history of hospitalization, surgery, venous thromboembolism, or medication use, and laboratory tests showed normal protein C and antithrombin III levels. There was no evidence of trauma, fracture, malignancy, chronic inflammatory disease, or antiphospholipid syndrome. However, since Lp(a) is a potential contributing factor to thrombus formation, it is noteworthy that his Lp(a) level was elevated at 60.6 mg/dL.

**Figure 2:**
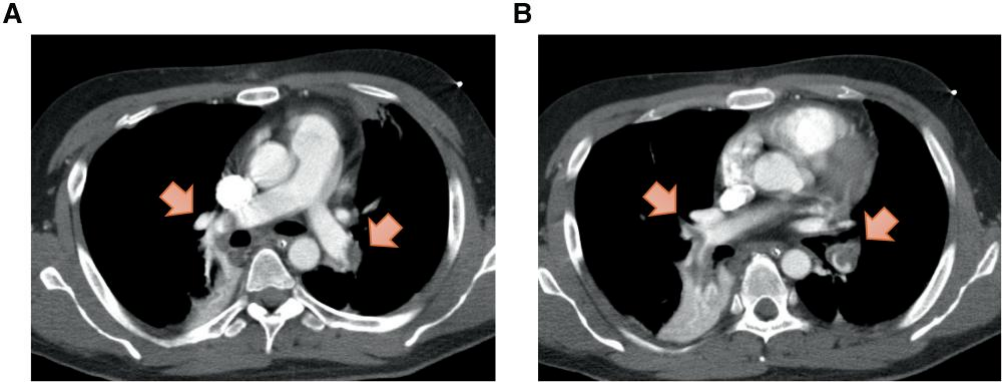
Findings on computed tomography images obtained on hospital Day 13. (*A*, *B*) Images showing newly developed thrombi in the right middle lobe and left lower lobe branches of the pulmonary artery (arrows).

**Figure 3:**
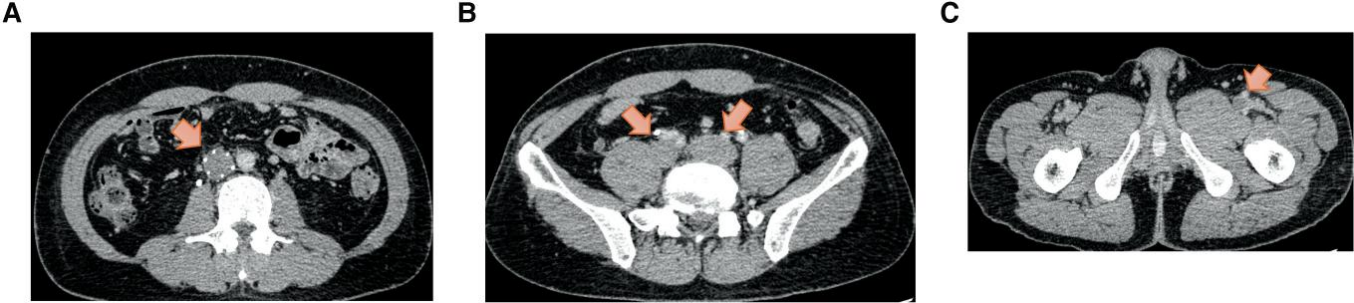
Findings on computed tomography images obtained on hospital Day 37. (*A*–C) Images showing a thrombus extending from the left femoral vein into the inferior vena cava filter (arrows) despite anticoagulation therapy with heparin.

Anticoagulation with unfractionated heparin was maintained at an aPTT 1.9–2.6 times the control value, slightly above the standard therapeutic range. Prior to weaning, the patient remained haemodynamically stable without vasopressor support (blood pressure 130/88 mmHg, heart rate 92 bpm), with normal arterial blood gas values (pH 7.416, pO_2_ 97.3 mmHg, pCO_2_ 40.0 mmHg, HCO_3_^−^ 25.1 mmol/L, lactate 1.1 mmol/L) and preserved right ventricular function (no septal flattening, RV/LV ratio 0.75, TAPSE 18.7 mm). Haemodynamics remained stable with heparin therapy, allowing successful discontinuation of VA-ECMO on day 8. On day 13, the patient developed tachypnoea and respiratory distress. Although the D-dimer level had decreased to 34 µg/mL, it subsequently increased again to 130 µg/mL. Repeat CT demonstrated newly developed thrombi in the right middle lobe and left lower lobe branches of the pulmonary artery and progression of thrombi in both superficial femoral veins (*Figure 2*). Although assessment was limited due to sedation and mechanical ventilation, the PESI score of 43 and sPESI score of 0 indicated a very low-risk category, and anticoagulation with unfractionated heparin was therefore continued. An IVC filter was placed because of thrombus propagation despite ongoing anticoagulation. The patient was extubated on Day 16 after stabilization of haemodynamic and respiratory conditions. Follow-up CT on Day 23 revealed near-complete resolution of pulmonary emboli with unfractionated heparin therapy; however, by Day 37, thrombi in the left lower extremity had extended into the inferior vena cava, filling the IVC filter with thrombus (*Figure 3*).

**Figure 4:**
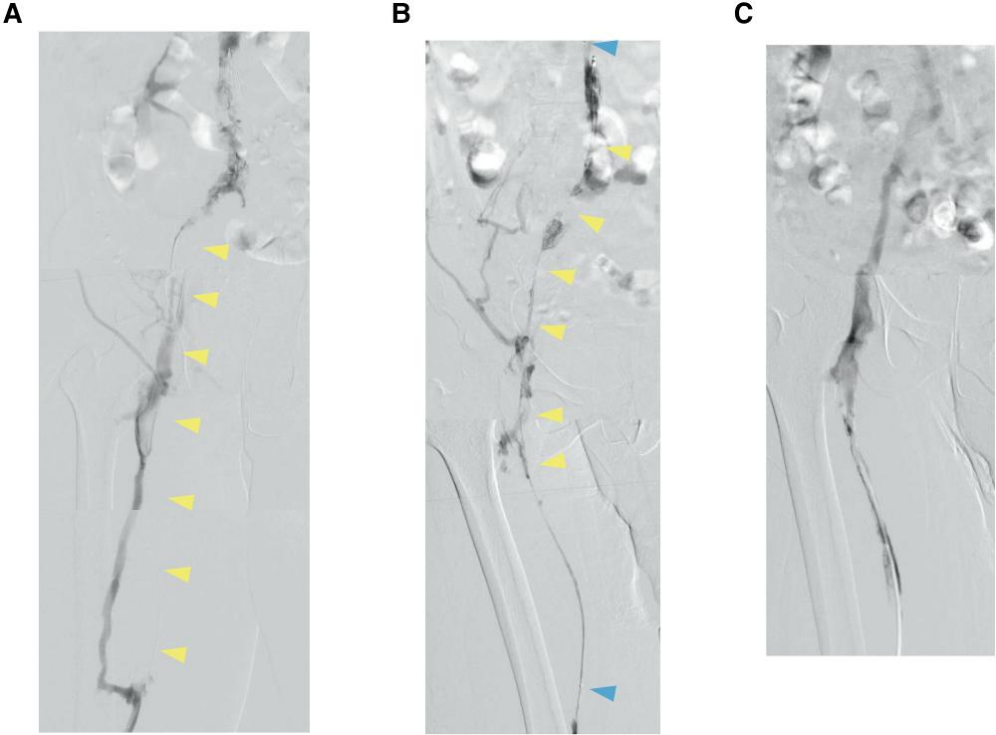
Findings on serial angiography examinations. (*A*) Angiography performed on Day 42 showed an occluded superficial femoral vein and collateral circulation via the deep femoral vein (arrowhead). (*B*) Angiography performed on Day 45 revealed residual thrombus in the iliac vein (arrowhead) despite continuous infusion of monteplase. The top and bottom arrowheads indicate both ends of the Fountain catheter placed across the inferior vena cava filter. (*C*) Angiography performed on Day 49 confirmed resolution of the thrombus in the iliac vein following administration of monteplase using the pulse-spray technique.

Treatment on the left side was prioritized in view of its larger thrombus burden. Angiography via the left popliteal vein revealed occlusion of the superficial femoral vein with collateral flow through the deep femoral vein (*Figure 4A*). Partial thrombectomy was achieved using an 8-Fr JR4 catheter. In view of the residual thrombus, a 4-Fr Fountain catheter with 50-cm side holes (Merit Medical Systems, Salt Lake City, UT, USA) was placed through the occluded portion of the filter using an Amplatz stiff guidewire. Monteplase (200 000 U) was administered through the catheter via pulse-spray, followed by continuous infusion of the same dose every 24 h for 3 days.

**Figure 5:**
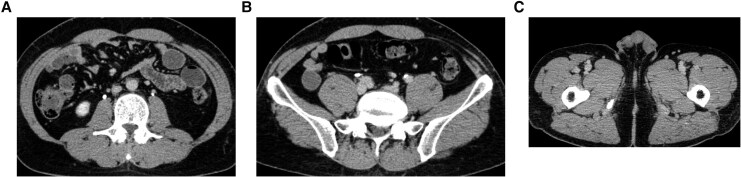
Computed tomography scans were obtained 7 weeks after removal of the inferior vena cava filter. (*A–C*) Images confirming complete resolution of thrombus (arrows).

Follow-up angiography after 3 days showed residual thrombus, so the dose of monteplase administered via pulse-spray was increased to 200 000 U twice daily (*Figure 4B*). Imaging performed 4 days later confirmed complete resolution of the thrombus in the left iliac vein. Balloon angioplasty was performed for the remaining femoral thrombus and restored autonomous blood flow (*Figure 4C*). The IVC filter was successfully removed on Day 52. Follow-up CT performed 7 weeks later in the outpatient setting showed complete resolution of the thrombus, and the D-dimer level had returned to normal (*Figure 5*).

## Discussion

This case highlights the value of pulse-spray CDT using monteplase, a tissue plasminogen activator (tPA), for recurrent PE and DVT when the IVC filter is occluded by thrombus. Monteplase was chosen because of ongoing shortages of urokinase. While monteplase is effective for PE, it carries a high risk of severe bleeding. Niwa *et al*. reported that the 30-day survival rate was 89.2% after systemic administration of monteplase but that severe bleeding complications occurred in 8.1% of cases.^2^ Unlike systemic thrombolysis, the pulse-spray technique can be used locally, administering small, frequent doses without significantly increasing the systemic drug concentrations and thereby reducing the bleeding risk.^3,4^

Serial monitoring of fibrinogen levels is recommended to minimize the risk of bleeding associated with administration of a tPA agent. Skeik *et al*. demonstrated that a fibrinogen level of <150 mg/dL is associated with an increased risk of major bleeding during tPA therapy.^5^ In the present case, serial fibrinogen monitoring was also performed during administration of monteplase to mitigate the bleeding risk further. However, there was no significant decrease in the fibrinogen level.

For local administration of monteplase via a Fountain catheter, methods include continuous infusion and the pulse-spray technique. With continuous administration, the drug is more likely to exit through the side hole, where resistance is least (i.e. a site free of thrombus). However, using the pulse-spray method, the drug can be administered directly to the thrombus because of the pressure applied, potentially allowing the thrombus to be dissolved more easily. Previous reports have also suggested that the pulse-spray CDT via a Fountain catheter improves dissolution of thrombus with less risk of bleeding.^6^ In this case, continuous administration of monteplase for 3 days dissolved only some of the thrombus. Therefore, we changed to the pulse-spray method. After 4 days of monteplase delivered by pulse-spray, the thrombus in the iliac vein was dissolved completely. Although the amount of monteplase administered and the duration of treatment increased, the pulse-spray method was considered more effective than continuous administration. Therefore, the use of the pulse-spray method and a Fountain catheter is more effective and safer in the event of thrombus progression.

In this case, blood tests did not reveal any underlying cause of venous thrombosis, and there was no evidence of predisposing factors, such as prolonged immobilization, rendering the aetiology unclear. However, the patient had elevated Lp(a) levels. Lp(a) is a lipoprotein involved in atherothrombosis, oxidative stress, and inflammatory responses and is a known risk factor for development of atherosclerotic cardiovascular disease, including myocardial infarction.^7^ However, there is some evidence suggesting that high Lp(a) is the one of possible contributing factor of thrombus formation.^8^ Genetic testing was performed, but no mutations were detected in the LPA gene that regulates Lp(a) production. Although elevated Lp(a) may be associated with thrombus formation, there is no specific therapy that can reduce Lp(a), and lifelong anticoagulation was deemed necessary.

## Conclusion

This case suggests that pulse-spray CDT using monteplase is useful for managing IVC filter-associated thrombosis, particularly when anticoagulant therapy is ineffective and filter occlusion is anticipated, enabling safe removal without bleeding complications.

## Data Availability

The data underlying this article will be shared on reasonable request to the corresponding author.

## Lead author biography

Atsushi Sato graduated from National Defense Medical College in Saitama, Japan, and subsequently completed his residency at National Defense Medical College Hospital. He then worked as a cardiologist in the Department of Cardiology at National Hospital Organization (NHO) Saitama Hospital in Saitama, Japan.

## Notes

**Consent statement:** The authors confirm that a written consent for submission and publication of this case report, including images and text, was obtained from the patient in line with COPE guidance.

## References

[1] Duffett L, Carrier M. Inferior vena cava filters. J Thromb Haemost 2017;15:3–12. 28019712 10.1111/jth.13564

[2] Niwa A, Nakamura M, Harada N, Musha T. Observational investigation of thrombolysis with the tissue-type plasminogen activator monteplase for acute pulmonary embolism in Japan. Circ J 2012;76:2471–2480. 22785619 10.1253/circj.cj-12-0091

[3] Goldhaber SZ, Magnuson EA, Chinnakondepalli KM, Cohen DJ, Vedantham S. Catheter-directed thrombolysis for deep vein thrombosis: 2021 update. Vasc Med 2021;26:662–669. 34606385 10.1177/1358863X211042930 PMC9009765

[4] Zhang RS, Maqsood MH, Sharp ASP, Postelnicu R, Sethi SS, Greco A, et al Efficacy and safety of anticoagulation, catheter-directed thrombolysis, or systemic thrombolysis in acute pulmonary embolism. JACC Cardiovasc Interv 2023;16:2644–2651. 37855802 10.1016/j.jcin.2023.07.042

[5] Skeik N, Gits CC, Ehrenwald E, Cragg AH. Fibrinogen level as a surrogate for the outcome of thrombolytic therapy using tissue plasminogen activator for acute lower extremity intravascular thrombosis. Vasc Endovascular Surg 2013;47:519–523. 23899656 10.1177/1538574413497107

[6] Cho KJ, Recinella DK. Pattern of dispersion from a pulse-spray catheter for delivery of thrombolytic agents: design, theory, and results. Acad Radiol 1997;4:210–216. 9084779 10.1016/s1076-6332(05)80293-4

[7] Hoogeveen RC, Ballantyne CM. Residual cardiovascular risk at low LDL: remnants, lipoprotein(a), and inflammation. Clin Chem 2021;67:143–153. 33257928 10.1093/clinchem/hvaa252 PMC7793228

[8] Konieczynska M, Natorska J, Zabczyk M, Undas A. Lipoprotein(a) and thromboembolism: current state of knowledge and unsolved issues. Arch Med Sci 2024;20:1770–1783. 39967936 10.5114/aoms/197357 PMC11831339

